# Self-Etch Silane Primer: Reactivity and Bonding with a Lithium Disilicate Ceramic

**DOI:** 10.3390/ma13030641

**Published:** 2020-01-31

**Authors:** Maria Dimitriadi, Spiros Zinelis, Maria Zafiropoulou, Nikolaos Silikas, George Eliades

**Affiliations:** 1 Department of Biomaterials, School of Dentistry, National and Kapodistrian University of Athens, 11527 Athens, Greece; mar.dimitriadi82@gmail.com (M.D.); szinelis@dent.uoa.gr (S.Z.); maria.zaf28@hotmail.com (M.Z.); 2 Dentistry, School of Medical Sciences, University of Manchester, Manchester M15 6FH, UK; Nikolaos.Silikas@manchester.ac.uk

**Keywords:** self-etch silane primer, reactivity, roughness, bond strength, lithium disilicate, NMR, IR

## Abstract

The aim of the study was to evaluate the stability, reactivity, and bond strength with a lithium disilicate ceramic of a self-etch silane primer (Monobond Etch and Prime/MEP). The stability was evaluated by ^1^H-,^31^P-NMR spectroscopy (before/after aging), and the reactivity by micro MIR-FTIR spectroscopy on Ge surfaces (0, 1, 24 h) using a prehydrolyzed silane primer (Calibra Silane Coupling Agent/CLB), as a control. The effect of MEP vs. 5% HF-etching on ceramic roughness was assessed by optical profilometry. The shear bond strength (SBS) of a resin composite bonded to polished ceramic surfaces treated with MEP, HF without silane (HF+NS), HF+CLB, and HF+MEP (n = 20) was evaluated after storage in water (A: 37 °C/1 week, B: 5000×/5–55 °C and C: 100 °C/24 h). Aging did not affect the silanol groups of MEP, but only the phosphate co-monomer. Silanols were reactive forming siloxanes, but exhibited lower consumption rate than CLB. HF-etching induced significantly higher values than MEP, in all the roughness parameters tested (Sa, Sz, Sdr, Sc, Sv), with the greatest differences found in Sdr and Sv. For SBS, MEP was inferior to all treatments/storage conditions, except of HF+NS in A, where the values were similar. However, on a HF-etched substrate, MEP provided highest strength and reliability.

## 1. Introduction

Acid-conditioning and silane priming of etchable glass-ceramic restorations have long been recognized as mandatory steps for a strong and durable bonding to tooth structure [1]. Despite the variety of glass-ceramics available, such as feldspathic, leucite-reinforced, lithium disilicate and resin-ceramic hybrids, acid-etching with hydrofluoric acid (HF, 5–10 wt%) and silanization with hydrolyzed γ-methacryloxypropyl trimethoxysilane (MPTMS) have been instructed for preparation of the bonding surfaces, prior to application of resin luting agents [1,2,3]. Initially, two-step silanes for chairside hydrolytic activation have been introduced. Later, they were replaced by single-step prehydrolyzed silane solutions, silane primers with conventional or adhesive resin monomers, and silane containing universal dental adhesives. For many of these developments, though, questions have been raised regarding the stability and reactivity of the silane component [4,5,6,7,8,9].

Recently, an alternative approach has been proposed by the introduction of a unique self-etching ceramic primer, where the etching agent and silane components are incorporated in the same vial, for simultaneous etching and silanization. The product introduced (Monobond Etch and Prime, Ivoclar Vivadent, Schaan, Liechtenstein) is free of the toxic HF acid, which has been replaced by a water/alcohol solution of tetrabutyl ammonium dihydrogen trifluoride (TADF) etchant. Based on the manufacturer’s documentation [10], the product contains additionally, a methacrylate phosphate monomer and a methacrylate functionalized silane (apparently MPTMS). Nevertheless, according to the safety data sheet of the product [11], the presence of another silane without methacrylate functionalization, the bipodal bis-triethoxysilyl ethane (BTSE) is described, as well. The performance of this self-etch silane primer, as supported by bond strength data, has been reported to be similar [12,13,14], slightly inferior [15], or significantly lower than conventional HF-etching and silanization [16,17,18,19].

The aim of the present study was to evaluate (a) the stability and reactivity of the silane components of the self-etch ceramic primer, (b) the roughness induced on the surface of a lithium disilicate glass ceramic by the acidic component of the primer, and (c) the bond strength of the primed lithium disilicate glass-ceramic with a resin composite. The null hypotheses were: The self-etch primer (a) does not retain adequate stability after aging and, as received, is not reactive to form siloxane polymers, (b) creates a rough surface similar to HF acid, and (c) provides high bond strength with the glass-ceramic, comparable to conventional HF-etching and silanization treatments.

## 2. Materials and Methods

The materials used in the study are listed in Table 1. The stability and reactivity of MEP silanes were investigated by NMR and IR spectroscopy, the effect on lithium disilicate surface roughness was evaluated by optical profilometry and the bond strength by a shear test.

### 2.1. Silane Stability

Two bottles of MEP were used as received and two after one month of aging (48 ± 1 °C) in a dry-heat oven. For the silane stability, aliquots of MEP were dried under a stream of Ν_2_ gas and the residue was dissolved in deuterated dimethyl sulfoxide (DMSO-d6). ^1^H- and ^31^P-NMR spectra were acquired on a spectrometer (Avance DRX 500 MHz, Bruker Biospin, Rheinstetten, Germany) equipped with a broad band probe under the following conditions: (a) ^1^H-NMR: ns (number of scans) = 32, sw (scan width) = 16 ppm, 90° pulse = 8 μs, T = 298 K; (b) ^31^P-NMR: ns = 200, sw = 160 ppm (–80 to 80 ppm), 90° pulse = 14 μs, T = 298 K. The DMSO-d6 signal at 2.5 ppm (^1^H) and phosphoric acid at 0 ppm (^31^P) were used for scale calibration. Spectra of a prehydrolyzed MPTMS (CLB) and of 10-MDP (Ivoclar Vivadent, Schaan, Liechtenstein), as received, were used as controls for ^1^H- and ^31^P-NMR analyses.

### 2.2. Silane Status and Reactivity

The status of the hydrolyzed silanols in MEP was further evaluated by monitoring the shift of the α–CH_2_ peak to the originally methoxylated Si atom of MPTMS [–CH_2_–Si– (OCH_3_)_3_] and ethoxylated Si atom of BTSE [–CH_2_–Si– (OCH_2_CH_3_)_3_] within the 11–0 ppm range of ^13^C-NMR spectra acquired as above, under the following conditions: ns = 6000, sw = 250 ppm, 90° pulse = 12 μs, T = 298 K, DMSO-d6 signal at 39.5 ppm for scale calibration. The α–CH_2_ peak has been chosen because of the widest splitting in comparison with the other CH_2_ peaks of the MPTMS chain. Therefore, it has been considered as the most suitable for monitoring the MPTMS hydrolysis process [20].

The silane condensation capacity in MEP was assessed by micro-multiple internal reflectance infrared spectroscopy (micro ΜΙR-FTIR). Aliquots of MEP were applied on the sampling surface of 45° edge Ge crystals (10 × 5 × 0.5 mm) of a micro MIR-FTIR accessory (Perkin-Elmer, Buckinghamshire, Bacon, UK) attached to an FTIR spectrometer (Spectrum GX, Perkin-Elmer, Buckinghamshire, Bacon, UK). The primer was brushed with a fiber-microbrush for 20 s, left intact for 40 s, rinsed with a strong air/water spray for 20 s and air-dried for 20 s. The prehydrolyzed MPTMS primer (CLB), used as control, was applied with a microfiber, left to react for 60 s and air-dried for 10 s. Spectra of the two primers were recorded immediately after (T0) and following 1 h (T1) and 24 h (T24) storage at 37 ± 1 °C and 70% RH. The spectra acquisition parameters were: 4000–700 cm^−1^ wavenumber range, 4 cm^−1^ resolution and 40 scans co-addition. The depth of IR penetration into the adsorbed films was estimated as to 0.6 μm at 1000 cm^−1^. To study the kinetics of silanol consumption during the 24 h testing period, the peaks of the 1000–850 cm^−1^ wavenumber range were curve fitted employing a Gaussian algorithm (peak area mode) at standard width/variable shape mode and 2% zero baseline. The net area ratios of silanol (Si–OH; 904 cm^−1^) to C=C vibrations (CH_2_=C– wagging; 940 cm^−1^, which do not change during condensation polymerization) were used to quantify the phenomenon. Peak fitting analysis was performed by PeakFit v.4.12 software (Seasolve, Framingham, MA, USA). All experiments were replicated in triplicate. 

### 2.3. Effect on Ceramic Surface Roughness 

Cylindrical glass-ceramic specimens (IPS e.max Press, Ivoclar Vivadent, Schaan, Liechtenstein, Ø = 5 mm, h = 10 mm) were prepared and embedded in epoxy resin (Caldofix-2 Kit, Struers, Bellarup, Denmark) leaving the top cyclical surface free. All specimens were polished with 300–2500 grit-size SiC papers in a grinding-polishing machine (Dap-V, Struers, Ballerup, Denmark) and ultrasonicated for 5 min in distilled water. Five specimen groups were prepared (n = 5/group), as calculated by power analysis (power = 0.8, a = 0.05, minimum detectable difference in means = 100, expected standard deviation residuals = 40, relative to Sa) and the polished specimen surfaces received the following treatments: (a) left intact (REF group); (b) etched with the HF-etching gel (IPS Etching Gel, Ivoclar Vivadent, Schaan, Liechtenstein), left to react for 20 s, rinsed with water for 20 s and dried with oil-free air for 10 s (HF group); (c) treated with MEP (20 s brushing, 40 s intact, 20 s water rinsing); (d) etched with the HF-gel and treated with CLB (applied with a microfiber, left to react for 60 s, air-dried for 10 s), and (e) etched with the HF-gel and treated with MEP. The specimens immediately after treatments c, d and e were rinsed with ethanol (10 ml) and air-dried (30 s), to remove the loosely bound fraction and as much of the adsorbed silane fraction as possible. All specimens were examined under an optical profiler (Wyko NT1100, Veeco, Tuscon, AZ, USA) equipped with a Mirau lens, at 40× magnification (113.3 × 148.5 μm^2^ analysis area), vertical scanning mode, 2% modulation and tilt correction. The surface roughness parameters measured were the amplitude parameter Sa (the arithmetic average of the absolute values of the surface height deviations measured from the best fitting plane), the amplitude parameter Sz (the 10 point height over the surface, representing the average difference between the 5 highest peaks and 5 lowest valleys), the hybrid parameter Sdr (the developed area due to the surface texture versus an ideal plane area ratio) and the functional parameters Sc (core void volume, the volume supported by the surface from 10% to 80% of the bearing ratio) and Sv (surface void volume, the volume the surface would support from 80% to 100% of the bearing ratio). Three measurements were randomly performed on each specimen and averaged to yield a representative value.

### 2.4. Bond Strength

Four groups of the lithium disilicate glass-ceramic specimens (n = 20/group) were prepared. The number of specimens was estimated by power analysis (power = 0.8, a = 0.05, minimum detectable difference in means = 5, expected standard deviation residuals = 4.5). A masking tape was placed at the central part of each specimen, leaving a free circular area (Ø = 3 mm) for bonding. The specimens of the first, second and third groups were acid-etched with the HF-gel, the fourth group was treated only with the MEP, whereas the third group was subjected to an additional treatment with MEP. The specimens of the 1st group received no further treatment serving as HF-etched controls, while the specimens of the 2nd group were treated with CLB. Cylindrical acetal molds (internal diameter = 3 mm, external diameter = 5 mm, height = 2 mm) were placed over the treated surfaces, filled with a single layer of the low viscosity composite and irradiated for 30 s with a LED light-curing unit (Radii Plus, SDI, Bainswater, Victoria, Australia, standard mode, 1.5 W/cm^2^ light intensity). The specimen groups produced (HF+NS: HF-etching without silane; HF+CLB: HF-etching plus CLB; MEP: only the self-etch primer; HF+MEP: HF-etching plus the self-etch primer) were stored in distilled water (storage A: 37 °C/1 week) without demolding. Two additional specimen series were prepared as above and stored in two different conditions (storage B: thermal cycling 5000×/5–55 °C, 20 s dwell time per water bath, 10 s transfer time; storage C: immersion in 100 °C water for 24 h), to assess the interfacial strength under these accelerated aging conditions promoting silane hydrolysis. All the specimens were then debonded under interfacial shear loading, with the notched-edge blade method [21] employing a universal testing machine (Tensometer 10, Monsanto, Swindon, UK) operated at 1.0 mm/min crosshead speed. The results of the shear bond strength (SBS) were expressed in MPa (N/mm^2^) by dividing the force at break by the nominal bonding surface area of the glass-ceramic specimens. A stereo-microscope (M80, Leica Microsystems, Wetlzar, Germany) at 10× magnification and a light microscope (DM 4000B, Leica Microsystems, Wetlzar, Germany) operated in reflection at 50× magnification were used to assess the failure mode of the debonded ceramic surfaces. Failure modes were classified as adhesive at the ceramic-composite interface (Type I), cohesive within the composite (Type II), mixed of type I and II (Type III) and cohesive within the ceramic (Type IV).

### 2.5. Statistical Analysis

The results of roughness parameters were statistically analyzed by one-way ANOVA and Tukey HSD multiple comparison tests. The SBS data were analyzed by a two-way analysis of variance (treatment mode and storage condition as independent variables) plus post-hoc multiple comparison tests. Furthermore the SBS data were subjected to Weibull analysis to determine the failure probability. The shape or modulus (*β*-parameter, defines the variability of the results by expressing the size distribution of the flaws), the scale or characteristic strength, (*η*-parameter or *σ*_0_, indicates the strength value for which the 63.2% of the sample size were debonded), and the strength at 5% failure probability (*σ*_0.05_) of the Weibull distributions were calculated. Finally, a chi-square test was used to assess the results of adhesive failure mode per treatment and storage condition. The statistical analyses for power, roughness, SBS and failure mode were performed by SigmaStat software (SigmaPlot v.12.5, Systat Software Inc, San Jose, CA, USA). For the Weibull analysis, Origin Lab software (v.9.1 SRO, Northampton, MA, USA) was used. In all cases, a 95% confidence level was selected (α = 0.05).

## 3. Results

### 3.1. Silane Stability

**Figure 1:**
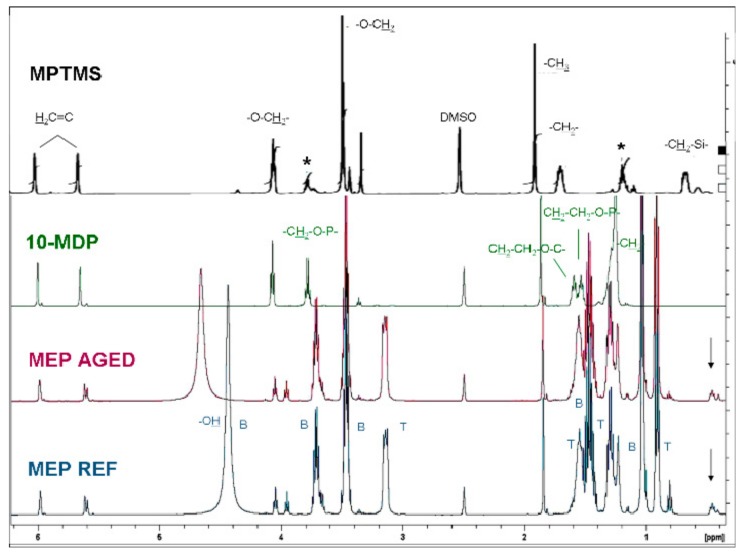
^1^H-NMR spectra of MEP, before and after aging, and of the controls (prehydrolysed MPTMS, 10-MDP). T and B peaks are assigned to TADF and butanediol, respectively. Arrows show the SiCH_2_CH_2_Si peaks of MEP and asterisks the ethoxylated derivatives in hydrolysed MPTMS.

**Figure 2:**
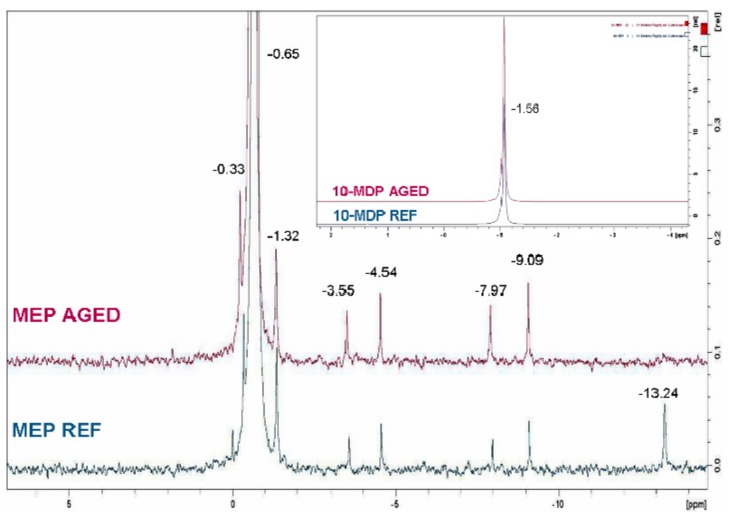
^31^P-NMR spectra of MEP and 10-MDP control (insert) before and after aging.

The ^1^H-NMR spectra of reference (REF) and aged MEP are illustrated in Figure 1. The peaks of TADF were identified at 3.16, 1.56, 1.30 and 0.93 ppm. The methyl doublet at 1.06 ppm indicative of a CHCH_3_ group, supports the presence of 1,3-butanediol (verified by ^1^H-^1^H TOCSY spectra). From the peaks assignable to the silane components, a weak singlet of the SiCH_2_CH_2_Si at 0.46–0.42 ppm was assigned to BTSE; the other peaks of this silane (expected at 3.8 and 1.15 ppm) [22] were masked off. The presence of MPTMS was not clearly confirmed in MEP; the CH_2_CH_2_Si group of the control (CLB) was not detected at 0.6 ppm. The same applies for the CH_2_CH_2_OP peaks of the phosphate monomer, which were overlapped by the strong peaks of TADF and solvents. However, the sets of methacrylate C=C protons (5.6 and 6.0 ppm), and the two methylene triplets (3.97 and 4.06) comply with the presence of MPTMS and/or a phosphate functionalized methacrylate ester. After aging the only changes observed were a small increase in the intensity of the 0.42 ppm peak and a small shift at lower ppm, possibly due to additional hydrolysis. The ^31^P-NMR spectra of MEP before and after aging are illustrated in Figure 2, along with the 10-MDP control.

A major peak attributed to O=P(OR)_3_ at −0.65 ppm dominated the MEP spectra, with several small peaks (<2% of total at −0.33, −1.32, −3.55, −4.54, −7.97, −9.09 and −13.24 ppm) suggesting the presence of various phosphoric ester phases (mono-, di-, tri-). After aging the peak at −13.24 ppm disappeared possibly due to broadening, but the others remained the same.10-MDP remained stable after aging. 

### 3.2. Silane Reactivity

**Figure 3:**
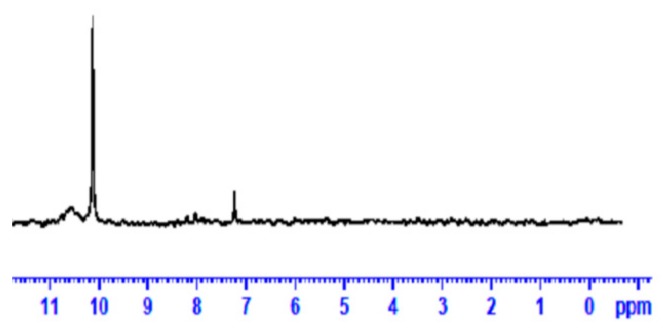
^13^C-NMR spectrum of MEP at 11–0 *δ* (ppm).

The ^13^C-NMR spectrum of MEP at the 11–0 ppm range is presented in Figure 3. The α–CH_2_ vibrations at 7.2 ppm are possibly assigned to di-methoxy siloxane dimers and at 10.1 ppm to condensates of MPTMS of the formula −CH_2_–Si–(OCH_3_)_3-n_(OH)_n1_(O–Si−CH_2_–)_n2_ with residual silanol activity [9,20]. No BTSE peaks were detected at this region, although expected at 2.2 ppm.

**Figure 4:**
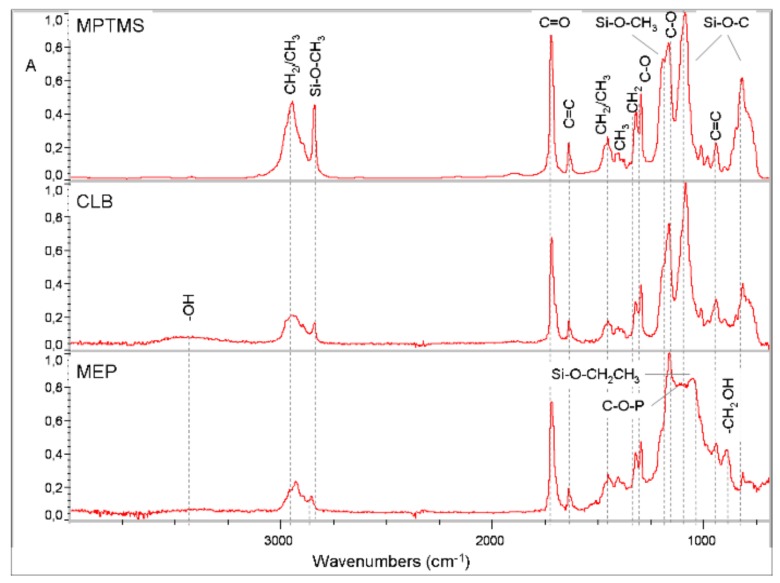
Micro MIR-FTIR spectra of MEP, CLB and of non-hydrolyzed MPTMS, immediately after application to the Ge crystals.

Representative micro MIR-FTIR spectra of MEP, CLB and of reference non-hydrolyzed MPTMS (Evonik Industries, Darmstadt, Germany), recorded immediately after application on the Ge crystals (T0) are illustrated in Figure 4. The peaks identified were (in cm^−1^): –OH (3460 in MEP, CLB), CH_2_/CH_3_ (2900–2800, 1450, 1400, in all), Si–OCH_3_ (2840, 1185, 1088, in all; the first shifted +10 cm^−1^ in MEP), C=O (1720, 1320, 1164, in all), C=C (1635, 940, 810, in all), Si–O– CH_2_CH_3_ (1049 cm^−1^, in MEP), CH_2_–O–P (1035, in MEP), CH_2_–OH (880, in MEP), and Si–OH (904, in MEP, CLB).

**Figure 5:**
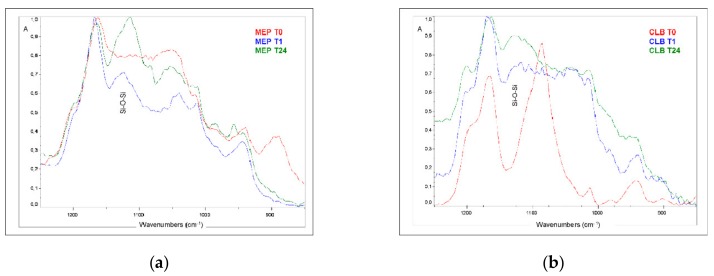
Micro-MIR FTIR spectra of MEP (**a**) and CLB (**b**) recorded immediately after application (T0), after 1 h (T1) and after 24 h (T24) storage (37 °C/70% RH). The developing Si–O–Si peaks due to the condensation reaction are clearly resolved (1250–850 cm^−1^ wavenumber range).

Expanded spectra of MEP and CLB at the 1250–850 cm^−1^ band range recorded at T0, T1, and T24 are illustrated in Figure 5. This range includes the silane absorption bands of methoxy (Si–OCH_3_; 1850, 1088 cm^−1^), ethoxy (Si–OCH_2_CH_3_; 1049 cm^−1^), siloxane (Si–O–Si; 1130–1000 cm^−1^), CH_2_=C– (940 cm^−1^), silanol (Si–OH; 904 cm^−1^) and ethanol related (–CH_2_–OH; 881 cm^−1^) groups [23]. At T1, new peaks of Si–O–Si antisymetric and symmetric vibrations appeared at 1130–1100 and 1050–1000 cm^−1^ respectively, which further increased in intensity at T24 implying the formation of condensates.

**Figure 6:**
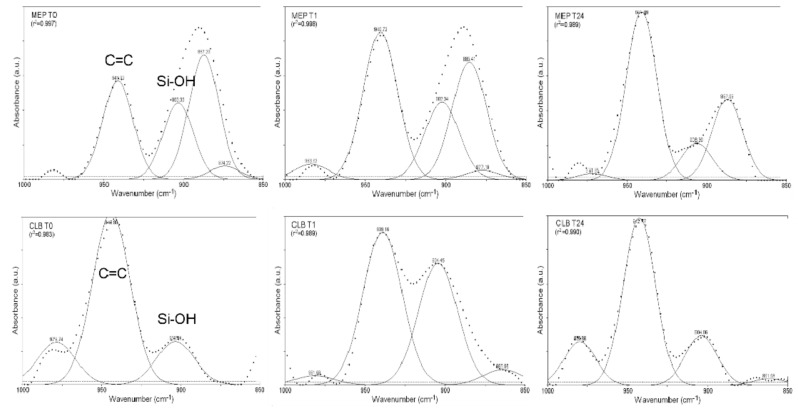
Gaussian curve-fitted spectra of MEP and CLB at T0, T1, and T24 time intervals, with the characteristic peaks of silanols (Si–OH, 904 cm^−1^) and C=C bond vibrations (CH_2_=C–, 940 cm^−1^), used for quantification of the silanol consumption (1000–850 cm^−1^ wavenumber range).

**Figure 7:**
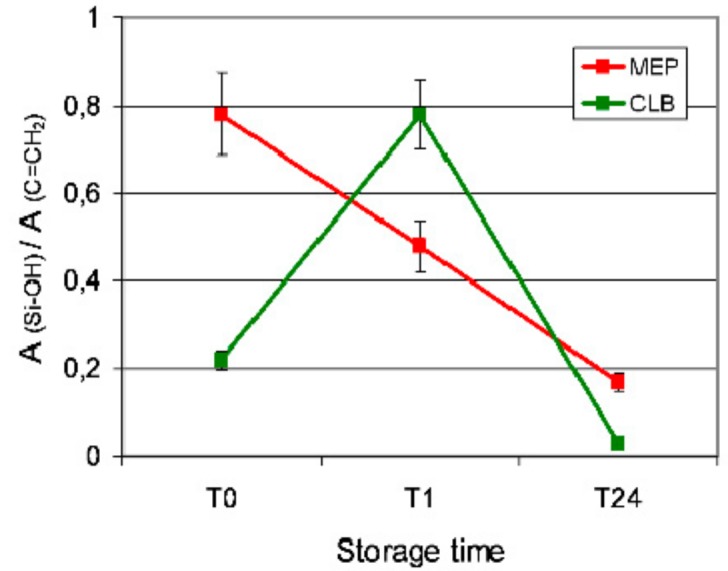
The normalized silanol consumption rate in MEP and CLB as a function of storage period.

The silanol consumption rate during the 24 h testing period was monitored by plotting the silanol (Si–OH, 904 cm^−1^) to the C=C (CH_2_=C–, 940 cm^−1^) peak area ratios obtained from the curve-fitted spectra of MEP and CLB (Figure 6). The results are presented in Figure 7. MEP demonstrated a reduction of the ratio as a function of time. However, CLB at 1 h (T1) demonstrated significantly higher ratio than the initial values (T0), which were then strongly reduced at 24 h (T24).

### 3.3. Effect on Ceramic Surface Roughness

**Figure 8:**
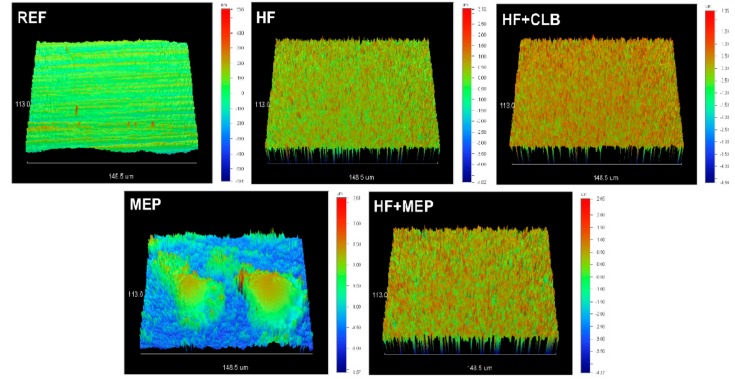
3D-profilometric images (40× magnification) of the polished lithium disilicate ceramic before (REF, 1.14 μm amplitude range) and after treatments with HF (8 μm amplitude range), HF+CLB (7.01 μm amplitude range), MEP (4.18 μm amplitude range), and HF+MEP (5.5 μm amplitude range).

3D-profilometric images of the lithium disilicate ceramic surfaces before and after treatments with the HF-gel, MEP and their combination are illustrated in Figure 8. The reference surface (REF), demonstrating the finishing and polishing tracks, became rough with a more homogeneous texture and an increase in the amplitude scale after HF-etching (HF). HF+CLB showed no difference from HF. Treatment with MEP illustrated a substantially reduced amplitude scale from the former, but still higher than the reference, with randomly distributed protruding domains. When MEP was used after HF etching (HF+MEP), the amplitude scale was higher than the reference and MEP, but lower than HF. The characteristic protruding domains of MEP were not identified in HF+MEP. The quantitative results of the roughness parameters tested are presented in Table 2. The rankings of the statistically significant differences were: HF, HF+CLB, HF+MEP >MEP>REF for Sa; Sz, Sv; HF, HF+CLB, HF+MEP>MEP, REF for Sdr and HF, HF+CLB>MEP>REF with HF+MEP possessing insignificant difference from HF, HF+CLB, MEP for Sc.

### 3.4. Bond Strength

The results of the descriptive statistics for SBS are presented in Table 3. Since equal normality tests failed, non-parametric statistics were used for the comparisons. 

The strong interaction (*p* < 0.001) between treatment mode and storage conditions dictated the use of separate Kruskal–Wallis ANOVA plus Tukey tests for comparisons of median values among the various treatment modes per storage condition and among the storage conditions per treatment mode. For storage condition A (37 °C/1 week) the ranking in statistical significance was HF+MEP, HF+CLB > HF+NS, MEP, whereas for conditions B (5000×/5–55 °C) and C (100 °C/24 h) were HF+MEP, HF+CLB > HF+NS > MEP (*p* < 0.05). For treatment modes the rankings were A, C > B for HF+MEP, and A > B, C for HF+CLB, HF+NS, and MEP (*p* < 0.05).

The results of the Weibull analysis for the reliability (*β*-parameter), characteristic strength (*η*-parameter or *σ*_0_) and strength at 5% failure probability (*σ*_0.05_) are summarized in Table 3. The statistically significant rankings on the reliability of the SBS values between the treatment groups per storage condition were: Insignificant difference for storage conditions A, C, and HF+MEP > MEP, with HF-CLB, HF+NS demonstrating insignificant differences from each group for condition B. The ranking of the storage conditions per treatment group were: No difference for HF+CLB and HF+NS; A, B > C for MEP; B > C for HF+MEP, with A demonstrating insignificant difference from B, C. The corresponding values for the *σ*_0_ between the treatment groups per storage condition were HF+MEP, HF+CLB > MEP, HF+NS for A and HF+MEP, HF+CLB > HF+NS > MEP for B, C, whereas for storage conditions per treatment group were A > C, B for all treatments. For *σ*_0.05_, the significant differences between treatment groups per storage condition were limited to B (HF+MEP > HF+NS > MEP with HF+CLB showing no difference from HF+MEP, HF+NS) and C (HF+CLB, HF+MEP, HF+NS > MEP). For the effect of storage conditions per treatment, statistically significant differences were identified only in MEP and HF+NS (A > B, C). 

**Figure 9:**
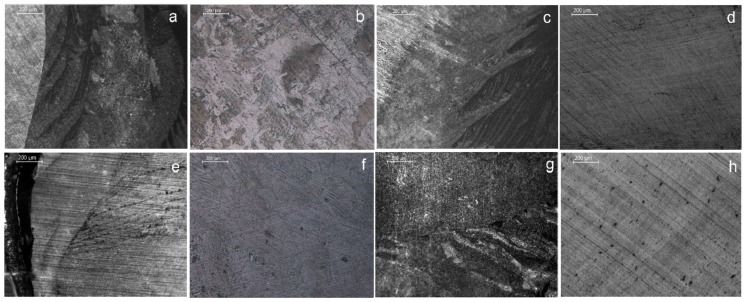
Representative reflected-light microscopic images of debonded ceramic surfaces (50×, bar = 200 μm) demonstrating Type III (mixed) failures with a great part of ceramic surface coverage by resin (**a**, **c**, **g**), Type III failures with minimal resin coverage (**b**, **e**) and Type I (adhesive) failures (**d**, **f**, **h**).

Representative microscopic pictures of debonded ceramic surfaces are illustrated in Figure 9. The results of the percentage adhesive failures are presented in Table 3. All other failures were mixed (Type III). No cohesive ceramic failure (Type IV) was identified. The percentage of adhesive failures was increased after thermal-cycling in all specimens, with the highest values observed in HF+NS and MEP. The chi-square test showed no statistically significant difference in the adhesive failure modes (chi-value: 1.052, *p* = 0.98).

## 4. Discussion

The results of the present study led to the rejection of the first null hypothesis; MEP demonstrated minimal changes after aging and exhibited reactive silanol groups capable of producing siloxane condensates. The second and third hypotheses should be rejected; the conventional 5% HF-etching treatment manifested higher roughness values on the lithium disilicate ceramic surfaces and in combination with a prehydrolyzed silane primer (CLB) provided greater resin bond strength than MEP.

MEP has been introduced as a self-etch silane-containing primer for etchable glass-ceramics. It is an aqueous solution of TADF, silanes and a phosphate monomer buffered to an acidic pH with butanol and 1,3 butanediol solvents, to maintain silanol reactivity and diminish condensation under storage [24,25]. There are two silanes in MEP: (a) The mono-silane MPTMS, a typical resin-ceramic coupling agent, to bond with silica and copolymerize with methacrylate based resin restorative material, and (b) the bipodal-silane BTSE, with two silane substitutions, to enhance the crosslinking capacity of the siloxane network formed. BTSE is classified as a “bridged” bipodal silane (silane substitution at head and tail of the molecule) without functional groups for co-polymerization with methacrylate-based resins; such groups may appear only in “pendant” bipodal silanes, where the substitution sites are separated by one or two carbon atoms (i.e., branched structures) [26]. Bipodal silanes when combined with organofunctional monosilanes retain the adhesive properties of the latter but with enhanced hydrolytic stability, being more efficient interfacial water barriers than monosilanes [27]. BTSE demonstrates high crosslinking density, due to the short length of the bridging group and hydrophobicity, assigned to the nature of the CH_2_ chain [28]. 

MPTMS and BTSE may produce derivatives with resin monomers containing polar groups (–OH, –COOH, –PO_4_), in the expense of their reactivity [8,9,29,30]. The NMR and IR analysis demonstrated that the MEP retains silanol reactivity after aging, although changes in the phosphorous phases occurred, possibly related to phosphate ester substitution. It is not known if this substitution affects the functionality of the phosphate methacrylate monomer, which is not the commonly used 10-MDP, according to the ^31^P-NMR analysis.

The ^1^H-NMR and IR spectra documented the presence of silanols in fresh and aged MEP. However, the ^13^C-NMR analysis showed that silanols may belong to more complex structures than fully hydrolyzed silanol monomers [9,20]. Under the conditions of the present study, the micro MIR-FTIR analysis verified that MEP is capable of forming siloxane bonds (intermolecular and with the substrate), a reaction that may proceed for more than 24 h due to the presence of residual silanols. MEP, with MPTMS and BTSE silanes, presented a different silanol consumption rate from the prehydrolyzed CLB control, which contains only prehydrolyzed MPTMS with approximately 40% residual methoxy groups, capable of further hydrolyzing in situ [8,31]. Although the extent of BTSE hydrolysis in MEP is unknown, identification of ethoxy groups at T0 implies incomplete BTSE hydrolysis in MEP. The increased normalized silanol peak area of CLB at T1 shows that the extent of the additional in situ hydrolysis of residual methoxy groups and the resultant silanol consumption rate are more pronounced than in MEP. This was expected since the methoxy groups of MPTMS hydrolyze faster than the ethoxy groups of BTSE [32]. In the present study, Gaussian curve fitting was performed to clearly resolve the silanol peak (904 cm^−1^), which is frequently confused with the peak of wagging CH_2_=C– vibrations of the methacrylate MPTMS moieties (940 cm^−1^). Normalized than simply peak Si–OH area ratios were used for quantification to minimize the error. However, in CLB there is a single source of Si–OH and C=C groups (MPTMS), whereas in MEP multiple sources exist (BTSE for Si–OH, methacrylated phosphoric acid ester for C=C and MPTMS for both). This difference may limit the reliability of direct comparisons per treatment period between the two silane primers. The siloxane consumption rates on the etched ceramic surface may be faster than on acid-insensitive Ge crystals due to increased amount of surface –OH groups from the surface protonization procedure after etching and water rinsing. Despite this limitation, internal reflection IR spectroscopy with Ge crystals, provides important information in submicron scale for the interfacial reactions (Si–O–Si and Si–O–Ge formation), with limited bulk film interferences [33].

The etching effect of MEP is mainly attributed to TADF, a well-established etchant for silica films [34]. However, the effect of MEP on surface roughness parameters was significantly lower than the 5% HF, as the pH of the former was higher. Both treatments were efficient in removing the polishing tracks of the control specimens, which were highly polished (2500 final grit-size) to improve the sensitivity of the changes in the roughness values induced. The roughness measurements were performed on the silanated substrates (HF+CLB, MEP, HF+MEP) after ethanol rinsing, to remove as much adsorbed silane as possible. For CLB, a pH of 5.5 has been reported [25], which is very weak to induce changes in surface roughness of intact or HF-etched specimens, as documented in the present study. However, considering the differences in the etching capacity between MEP (pH = 3.7) and HF (pH = 2), it is reasonable to anticipate variations in the surface roughness, due to limited removal of the glassy ceramic phase in MEP [15], which created the protruding domains or residual glass and surface texture heterogeneity observed. The lower pH of the HF demonstrated two times greater Sa, Sz, 18 times greater Sdr, 1.5 times greater Sc and four times greater Sv values than MEP. HF-etching created a high surface area (Sdr) topography, with a major contribution of deep valleys (Sv) in comparison with MEP. These findings indicate that the commonly used amplitude parameters Sa and Sz may not reliably express the magnitude and quality of the surface roughness changes induced by the etchants, relevant to ceramic adhesion. The roughness parameters recorded on HF+MEP surfaces were statistically insignificant from HF, but with higher standard deviations at lower mean values for Sa, Sz, Sc, and percentile range median values for Sdr, indicating greater variance of the MEP treatment effect on HF-etched surfaces, which resembles the corresponding performance of MEP. Possibly the unfavorable surface wettability and spreading coefficient values of MEP documented for leucite-reinforced feldspathic and lithium disilicate ceramics may explain this finding [19]. 

The reduced surface roughness of the TADF-etched lithium disilicate specimens, raises a question on the capacity of MPTMS and BTSE to counterbalance the lower micromechanical retention, by establishment of strong chemical bonding via the two silanes. It has been postulated that the synergy of MPTMS with BTSE may improve the properties of the film formed especially under wet conditions [27]. Based on the SBS data, MEP treatment provided values comparable with HF+NS after storage in water at 37 °C /1 week. This suggests that for this primer the reduced micromechanical retention and dual-silane treatment were equally efficient with the micromechanical retention provided by the HF, without any silane treatment. However, after accelerated aging (thermal-cycling, 100 °C/24 h), MEP resulted in the lowest values, below the threshold of the silane-free treatment. Storage of silane treated interfaces in hot or boiling water has been originally proposed by Pluedemmann [35], several decades ago, as a fast method for testing silane hydrolytic stability and has been implemented in studies of resin bonding to silane-treated surfaces [36,37], and silane-treated fillers and fibers [38,39,40,41]. Thermal cycling has officially been advocated as a means of accelerated aging in bond strength studies [21], although the claimed simulation of intraoral conditions is questionable, since intermediate isothermal relaxation periods are missing. Under the current experimental conditions, the lack of significant differences between thermal-cycling and hot water immersion implies that the latter may be used as the fastest aging procedure.

The results of accelerated aging tests confirmed that the increased surface roughness, apart from being a critical factor for micromechanical retention, it may preserve the silane bonding capacity, as manifested by the significantly higher values of HF+CLB and HF+MEP from HF+NS. It has been postulated that the stability and protective capacity of a silane film to environmental stimuli is dependent on chemical reactivity, hydrophobicity, extent of network cross-linking and film thickness [42]. Apparently, the increased ceramic protonated surface area after HF treatment, promoted in situ silane hydrolysis, bonding with the substrate and intramolecular condensation, reducing thus the detrimental effects of aging. It is interesting that, although there were statistically insignificant differences between HF+CLB and HF+MEP, the latter with the highest silane crosslinking capacity induced by BTSE, demonstrated a trend for higher values under all storage conditions. An additional factor that may account for the positive effect of HF+MEP is the water rinsing and air-drying stage after MEP application, which may remove more effectively the loosely bound fractions of MPTMS and BTSE from the rough HF-etched surface, reducing silane film thickness in favor of the chemisorbed components [43]. The silane film thickness has long been considered an important issue related to the interfacial strength, with a monolayer being the best molecular arrangement. Nevertheless, the optimum silane film thickness has been reported to vary from 50 to 150 nm, being much more than a monolayer [44]. For hydrolyzed MPTMS the film thickness ranged from 30–40 nm after air-drying, as measured on ultra-smooth electropolished silica surfaces, and was further reduced to 10–15 nm after hot water rinsing [43]. Such conditions may not apply on surfaces possessing high core and surface void volume (i.e., after HF etching), where silane retention is increased, differential solvent evaporation may occur and intermolecular condensation is promoted within deep valleys. In the presence of bipodal silanes, this may assist, to a certain extent, formation of highly crosslinked intermolecular structures for enhanced hydrolytic stability. Comparison of HF with HF+CLB and HF+MEP demonstrated insignificant differences in all roughness parameters tested indicating that CLB and MEP after ethanol rinsing have a negligible retention capacity on the very rough HF-etched substrate. Consequently, there is no reason to expect greater retention capacity of MEP silanes on a much smoother surface created by its self-etching capacity, after ethanol rinsing. Therefore, the possibility of implication of silane adsorption on roughness measurements, especially on nm-scale sensitive parameters such as Sa, is diminished. 

For clinical applications the 5% failure probability level (*σ*_0.05_) is considered as more relevant than the 63.2% level expressed by *σ*_0_ [45], as it may better clarify early failure incidence. In all treatments after 37 °C/1 week storage the *σ*_0.05_ values were >50% of *σ*_0_, except of HF+MEP (46% of *σ*_0_). However, after accelerated aging only HF+CLB, HF+MEP (thermal-cycling) and HF+CLB (100 °C/24 h) demonstrated values >50% of *σ*_0_. Consequently, only for HF+CLB the strength at 5% failure probability exceeded the 50% of the characteristic strength, indicating a more stable interface. The frequency of adhesive failures was increased after accelerated aging due to hydrolytic degradation. The statistically insignificant differences among the treatments may be appended to the fact that the effect was not fully validated, as the extent of resin coverage of the bonded ceramic surface was not quantified.

In the present study a non-adhesive low viscosity light-cured composite was used as the adherent material to match the viscosity of resin luting agents and adhesive cements used in clinical practice, but with higher flexural strength and fracture toughness [46] to reduce bulk composite fractures during shear loading, from the inevitable bending moments produced [47]. The non-adhesive nature was preferred in order to reveal the net contribution of the ceramic treatments (etching/silanization) to the interfacial strength rather than the combined effect of these treatments with the adhesive monomers incorporated in bonding agents and resin cements, which may modify the overall interfacial strength depending on their composition. A conventional macro-shear test was selected for the study, considering that micro-shear tests are not suitable for cases where low bond strength values are anticipated [48], as confirmed for MEP after accelerated aging. Shear tests and the specimen design used (bonded composite to a fixed ceramic) have been heavily criticized since they create subsurface critical tensile stresses and cohesive failures in the ceramic, which is actually loaded distal to the interface as determined by finite element analysis [49]. However, the development of much stronger ceramics than the feldspathic one used in the previous study certainly modifies the failure mode, minimizing cohesive ceramic failures. Despite the criticism, shear tests offer important information, especially when performed on a comparative basis, being very simple in design, easily executed by loading a single interface, applicable to many substrates and quite reproducible. These are the only tests that have been used in massive assessment of dental adhesives, leading to important conclusions for handling, technique sensitivity, and operator training issues [50,51]. The introduction of the notched-edge design of the loading element, improved the uneven stress distribution of the original knife-edge geometry, and was validated as a relevant international standard [20]. Finally, to face the limitations of brittle fracture behavior of the bonded composite and the related data scattering problems, Weibull statistics were additionally employed in the present study, expressing the failure probability. This method has been indicated as the best for bond strength data interpretation [52]. The results of the present study show that the bond strength of a resin composite with a lithium disilicate glass-ceramic, as mediated by the self-etch primer MEP, containing TADF etchant, a functional silane (MPTMS), a crosslinking silane (BTSE), and a phosphate monomer, was inferior under all storage conditions from the same functional silane applied on a conventionally (5% HF) etched substrate. However, the bond strength values of MEP showed no statistically significant difference from the conventional treatment when applied on HF etched ceramic surfaces. Although clinical studies are required to reveal the credibility of the findings of the present study, it can be concluded that ceramic surface roughness plays the pivotal role in strength and durability of resin bonding to the etchable glass-ceramic tested. Therefore, new developments, aiming to simplify treatment steps, should highly consider micromechanical retention capacity, especially when chemical bonding is susceptible to hydrolytic degradations, as in the case of silanes.

## 5. Conclusions

Based on the results of the present study, the following conclusions can be reached:

The self-etch silane primer was stable, retaining the original silanol activity after aging. However, the stability of the phosphate co-monomer was affected. 

The silanol consumption rate of the functional (MPTMS) and crosslinking (BTSE) silanes in the self-etch primer was different from that of the same functional silane alone; prehydrolyzed MPTMS demonstrated increased in situ hydrolysis and faster silanol consumption rate.

The self-etch silane primer resulted in significantly lower amplitude (Sa, Sz), hybrid (Sdr) and functional (Sc, Sv) roughness parameters in comparison with 5% HF-etching, when applied on polished lithium disilicate ceramic surfaces. The greatest differences were found in Sdr (18×) and Sv (4×) in favor of the HF acid. By using the self-etch primer on a HF-etched substrate, the values recorded for all the parameters increased at the levels provided by the HF acid.

The bond strength of a resin composite to the lithium disilicate as mediated by the self-etch primer was lower from that of the functional silane (MPTMS) and of the same primer applied on HF-etched surfaces, under all storage conditions. Accelerated aging strongly affected the self-etch primer group, leading to values even lower than the negative control (HF-etched substrate without silane).

## Figures and Tables

**Table 1:** The products used in the study.

Product	Composition *	Manufacturer
**1. Ceramic**		
IPS e.max Press	SiO_2_, Li_2_O, K_2_O, P_2_O_5_, ZrO_2_, ZnO, Al_2_O_3_, MgO, La_2_O_3_	Ivoclar Vivadent, Schaan, Liechtenstein
**2. Etchant**		
IPS Ceramic Etching Gel	4.9% HF acid, water, colorant (pH = 2)	Ivoclar Vivadent, Schaan, Liechtenstein
**3. Silane**		
Calibra Silane Coupling Agent (code: CLB)	MPTMS, ethanol, acetone	Dentsply Caulk Milford, DE, USA
Monobond Etch and Prime (code: MEP)	TADF, silane methacrylate, BTSE, methacrylated phosphoric acid ester, butanol, water, colorant (pH = 3.7)	Ivoclar Vivadent, Schaan, Liechtenstein
**4. Resin composite**		
G-aenial Flo Universal (A2 shade)	Resin: UEDMA, TEGDMA, BisEMA Filler: Silanated 0.2 μm Sr-glass, 16 nm SiO_2_ (69 wt%, 50 vol%)	GC Corp., Tokyo, Japan

* According to the manufacturers’ information. MPTMS: γ-methacryloxypropyl trimethoxysilane, TADF: Tetrabutyl ammonium dihydrogen trifluoride, BTSE: Bis(triethoxysilyl)ethane, BisGMA: Bisphenol-A glycidyl dimethacrylate, TEGDMA: Triethyleneglycol dimethacrylate, BisEMA: Ethoxylated bisphenol-A dimethacrylate.

**Table 2:** Results of roughness measurements *.

Treatment	Sa (nm)	Sz (μm)	Sdr (%)	Sc (nm^3^/nm^2^)	Sv (nm^3^/nm^2^)
REF	81.2 ^a^ (17.6)	0.7 ^a^ (0.1)	1.5 ^a^ (0.9–1.7)	112.4 ^a^ (24.5)	13.3 ^a^ (3.7)
HF	425 ^b^ (30.9)	4.5 ^b^ (0.4)	106.1^b^ (100.5–133.1)	537.2 ^b^ (68.5)	80 ^b^ (13)
HF+CLB	398 ^b^ (41.4)	3.9 ^b^ (0.6)	94.6^b^ (86.5–144.6)	510.8 ^b^ (83.7)	85.8 ^b^ (21)
MEP	184.2 ^c^ (73.7)	2.3 ^c^ (0.4)	5.6 ^a^ (3.1–6.7)	368.1 ^c^ (93.9)	21.6 ^c^ (4.7)
HF+MEP	363.3 ^b^ (107.5)	3.7 ^b^ (0.2)	71.6^b^ (56.7–101.2)	462 ^b,c^ (162.9)	69.1 ^b^ (9.8)

* Means and standard deviations for all parameters except of Sdr (median and 1st, 3rd percentiles), where non parametric analysis was used (Kruskal–Wallis) due to failure in normality test. Same superscripts show mean values with no statistically significant differences per roughness parameter (*p* > 0.05).

**Table 3:** Results of the shear bond strength test and percentage of adhesive failures *.

Treatment	Median (25%–75% percentiles) MPa	Weibull *β* (95% C.I.)	Weibull *σ*_0_ (95% C.I.) MPa	Weibull r^2^ (*η*)	Weibull *σ*_0.05_ (95% C.I) MPa	Adhesive Failures n, (%)
**Storage A: 37 °C/1 week**						
HF+CLB	31.5 ^a,A^ (23.5–38)	4.4 ^a,A^ (3.2–6.3)	34 ^a,A^ (30.6–37.7)	0.93	18.1 ^a,A^ (11.6–23.5)	4 (20)
MEP	20.4 ^b,A^ (16.9–25.3)	4.2 ^a,A^ (3–5.8)	23.6 ^b,A^ (21.1–26.3)	0.94	12.4 ^a,A^ (7.8–16.1)	8 (40)
HF+MEP	37.6 ^a,A^ (23–44.4)	4 ^a,A^ (2.8–5.8)	39.7 ^a,A^ (35.4–44.5)	0.91	18.1 ^a,A^ (10.3–25.4)	5 (25)
HF+NS	20.6 ^b,A^ (16.1–23.1)	4.6 ^a,A^ (3.3–6.4)	22.5 ^b,A^ (20.3–24.9)	0.91	12.8 ^a,A^ (8.7–16.2)	6 (30)
**Storage B: Thermal cycling (5000×/5–55 °C)**						
HF+CLB	18.4 ^a,B^ (14.9–22.5)	4.6 ^a,b,A^ (3.1–7)	20 ^a,B^ (17.8–22.6)	0.98	10.1 ^a,c,A^ (5.6–14.2)	9 (45)
MEP	3.6 ^b,B^ (3–6)	2.4 ^a,A^ (1.6–3.6)	5 ^b,B^ (4–6.2)	0.93	1.5 ^b,B^ (0.5–2.7)	14 (70)
HF+MEP	19.1 ^a,B^ (16.8–21.2)	7.4 ^b,A^ (5–11.1)	27.5 ^a,B^ (25.4–29.7)	0.94	13.6 ^a,A^ (9.8–16.5)	7 (35)
HF+NS	8.6 ^c,B^ (7.4–10.7)	3.8 ^a,b,A^ (2.5–6)	10.7 ^c,B^ (9.1–12.8)	0.95	4.2 ^b,c,B^ (1.9–7.4)	16 (80)
**Storage C: 100 °C/24 h**						
HF+CLB	21.1 ^a,B^ (17.8–29.3)	3.5 ^a,A^ (2.4–4.9)	25.6^a,B^ (22.5–29.2)	0.94	11.4 ^a,A^ (6.5–16.2)	7 (35)
MEP	5.1 ^b,B^ (3.8–6.3)	2.1 ^a,A^ (1.5–2.8)	6.1 ^b,B^ (4.9–7.6)	0.97	1.7 ^b,B^ (0.7–5)	15 (75)
HF+MEP	28 ^a,A^ (19.8–30.4)	2.6 ^a,A^ (1.9–3.7)	29.5 ^a,B^ (24.7–35.1)	0.92	10 ^a,A^ (6.9–15.1)	8 (40)
HF+NS	12.2 ^c,B^ (9.6–16.7)	2.9 ^a,A^ (2.1–4.1)	14.7 ^c,B^ (12.4–17.3)	0.94	5.6 ^a,B^ (2.8–8.4)	12 (60)

* Same superscripts show mean values with no statistically significant differences (p>0.05) between treatments per storage condition (lower case) and between storage conditions for the same treatment (upper case).
